# Does Integrating Cognitive and Psychological Interventions Enhance Wellbeing After Acquired Brain Injury? Study Protocol for a Phase II Randomized Controlled Trial of the VaLiANT (Valued Living After Neurological Trauma) Group Program

**DOI:** 10.3389/fresc.2021.815111

**Published:** 2022-01-21

**Authors:** Nick Sathananthan, Eric M. J. Morris, David Gillanders, Lucy Knox, Bleydy Dimech-Betancourt, Bradley J. Wright, Roshan das Nair, Dana Wong

**Affiliations:** ^1^School of Psychology and Public Health, La Trobe University, Melbourne, VIC, Australia; ^2^School of Health in Social Sciences, University of Edinburgh, Edinburgh, United Kingdom; ^3^Institute of Mental Health, University of Nottingham, Nottingham, United Kingdom

**Keywords:** cognitive rehabilitation, psychological therapy, acquired brain injury, valued living, Acceptance and Commitment Therapy, holistic rehabilitation, combined interventions

## Abstract

**Background and Objectives:**

Cognitive and emotional changes affect the majority of individuals with acquired brain injury (ABI) and are associated with poorer outcomes. The evidence for “siloed” rehabilitation approaches targeting cognition and mood separately remains mixed. Valued living (i.e., acting consistently with personal values) is associated with better psychological functioning and participation in work and other productive activities. Rehabilitation interventions that concurrently address cognitive and emotional barriers to valued living may therefore result in improved outcomes. VaLiANT (Valued Living After Neurological Trauma) is an 8-week group intervention developed by our team, which uniquely combines cognitive rehabilitation and psychological therapy to improve wellbeing and meaningful participation (i.e., valued living) following ABI.

**Method:**

This protocol describes the design and implementation of a Phase II parallel-group randomized controlled trial with blinded outcome assessors, to evaluate the potential efficacy of VaLiANT and the feasibility of a Phase III trial. Participants are adults with a history of ABI at least 3 months prior to study entry, who experience cognitive and/or emotional difficulties and associated reduced participation in valued activities. Random allocation to the treatment condition (8-week VaLiANT group program) or a usual care waitlist control condition occurs at a 2:1 treatment: control ratio. The primary outcome is wellbeing, measured by the Warwick-Edinburgh Mental Wellbeing Scale. Secondary outcomes include measures of valued living, mood, cognitive complaints, quality of life, community participation, post-traumatic growth, and self-efficacy. All measures are collected across three time points by blinded assessors (baseline, 8-week follow-up, 16-week follow-up). Trial feasibility will be evaluated against recruitment rates, drop-out rates, intervention acceptability, and treatment fidelity (manual adherence and therapist competence).

**Discussion:**

This trial will extend current knowledge on how to improve long-term outcomes following ABI by evaluating an innovative integrated, multi-domain approach to rehabilitation concurrently addressing cognitive and emotional barriers to participation in meaningful life roles.

## Introduction

Acquired brain injuries (ABIs) such as stroke and traumatic brain injury (TBI) frequently result in cognitive and emotional changes. Estimates suggest that over half of those with a TBI or stroke experience long-term cognitive impairment, especially in the domains of attention, memory, and executive functions (1, 2). Similarly, clinically significant levels of depression and anxiety affect one-third of stroke and half of TBI survivors (3–5) and rates of suicide following ABI are notably higher (6). These cognitive and emotional difficulties are interrelated and highly comorbid after ABI (7), with higher mood symptoms predicting increased cognitive complaints (8), and increased cognitive complaints predicting higher mood disturbance (9). Cognitive and emotional sequelae are frequently highlighted as areas of long-term unmet need by people with ABI, indicating that they are not managed adequately by existing services (10, 11).

Importantly, cognitive impairment and mood disturbance are associated with poor long-term outcomes following ABI. Cognitive impairment predicts reduced independence in activities of daily living (ADLs), reduced participation in meaningful life activities, and poorer overall quality of life (12–15). Furthermore, cognitive impairment is a stronger predictor of negative outcomes and overall disability at 5–10 years post-ABI than the initial injury severity (16, 17). Mood symptoms also predict reduced independence in ADLs, participation in meaningful life activities (18–21), and poorer quality of life (22, 23). As such, cognitive impairment and mood disturbance act as significant barriers to adjustment and recovery from ABI, highlighting the need for evidence-based interventions that address these difficulties.

Current treatment approaches typically remain domain-specific and target cognitive impairment or mood symptoms in isolation, with a limited focus beyond the impairment level (24). Evidence for these approaches remains inconclusive, with studies demonstrating variable efficacy and limited generalization to broader outcomes. For example, memory interventions tend to result in moderate improvements to both subjective and objective memory performance following ABI (25) but provide mixed findings regarding improvement in long-term functional outcomes and quality of life (26–28). Interventions targeting attention deficits have resulted in limited improvement to attention immediately following interventions with no generalization to other long-term outcomes (29). Cognitive Behavioral Therapy (CBT) can improve depressive and anxiety symptoms and some functional outcomes following stroke (30) but these effects have not been consistently found after TBI (31), although adapted CBT that incorporated cognitive compensatory strategies including follow-up booster sessions has shown promise for treating anxiety and depression following TBI (32) with associated improvements in psychosocial outcomes (daily functioning, work, relationships, leisure). Therefore, existing “siloed” treatment approaches do not consistently demonstrate improvements to mood or cognition and positive intervention effects do not consistently translate into improved long-term outcomes such as quality of life or participation in meaningful activities.

It then follows that cognitive rehabilitation and psychological therapy techniques may need to be integrated to holistically improve outcomes beyond the impairment level by concurrently targeting cognitive and emotional barriers to activity and participation in meaningful life roles, wellbeing, and quality of life (33, 34). There is a growing body of evidence suggesting that integrated rehabilitation interventions that combine both psychological and cognitive elements into broader frameworks lead to improvements in psychological distress, meaningful participation, and quality of life, with stable or ongoing improvement up to 3 years following treatment (35, 36). Randomized controlled trial (RCT) level evidence has also suggested that such approaches are more effective at improving outcomes than standard neurorehabilitation and traditional neuropsychological intervention (37, 38). Patients have described experiencing holistic neurorehabilitation as empowering and beneficial for everyday functioning (39). However, several challenges continue to limit the implementation of such interventions including a lack of funding, resources, or other systemic factors (34). The aforementioned interventions were all lengthy with a high frequency of sessions [e.g., 15 h per week over 16 weeks; (37)] which may not be easily implemented or appropriate for all health-care systems. Further research is needed to determine whether the positive effects of integrated, holistic interventions can be replicated when the length of intervention is briefer, which may be more cost-effective and more readily implemented into existing services.

Valued living refers to the extent to which we engage in behaviors that are consistent with our personal values, and it has gained growing attention as an important outcome post-ABI. Higher levels of valued living have been linked with improved wellbeing, quality of life, better psychosocial functioning, and lower psychological distress in both ABI (40, 41) and other chronic health condition populations (42–44). Valued living has been directly related to the level of acceptance and adjustment toward one's ABI (45). However, valued living often remains reduced for a number of years following brain injury (40). Rehabilitation interventions that target valued living may result in improved outcomes. Acceptance and Commitment Therapy (ACT) is an evidence-based psychological therapy that directly targets valued living, with growing evidence supporting its use to improve mood symptoms and psychological distress in TBI (46–49), stroke (50–52) and other neurological conditions (53, 54). However, none of these studies have specifically aimed to address cognitive impairment, and all have demonstrated limited impact beyond the level of mood disturbance and psychological distress.

A holistic and integrated intervention that targets both cognitive and emotional barriers to valued living may result in more consistent improvements to impairments (e.g., cognitive complaints or mood symptoms) while also leading to more global improvements in meaningful participation, wellbeing, and quality of life. Valued Living After Neurological Trauma (VaLiANT) is a new 8-week group intervention that aims to enhance adjustment to life with ABI by combining cognitive rehabilitation and psychological therapy using ACT principles. A Phase I study has been completed using a single case experimental design repeated across eight participants (55). This study demonstrated reliable improvements to a broad range of outcomes for the majority of participants, including overall wellbeing, anxiety symptoms, and subjective cognitive complaints. The delivery of the intervention was deemed feasible and participant acceptability ratings of the intervention were high. These Phase I findings suggested that VaLiANT may have utility in improving outcomes following ABI and warrants further investigation of the intervention.

Here, we report the protocol for our Phase II RCT evaluating VaLiANT, which aims to:

1. Compare the impact of VaLiANT against treatment-as-usual waitlist control on a range of adjustment-related outcomes including at the levels of impairment, activity, participation, and overall wellbeing and quality of life. This will identify signals of efficacy and determine parameter estimates for a definitive Phase III trial.
2. Investigate the feasibility of the trial design, including recruitment rate, retention rate, success of blinding the outcome assessor under RCT conditions, and exploring the fidelity of delivering the intervention.

## Method

### Ethics

This study has been approved by the La Trobe University Human Research Ethics Committee (HEC #18423) and has been registered in the Australian New Zealand Clinical Trials Registry (ACTRN12619001243101). Protocol amendments have been submitted to both bodies following methodological changes due to the impact of COVID-19. Written informed consent will be obtained from all participants.

### Study Design

**Figure 1:**
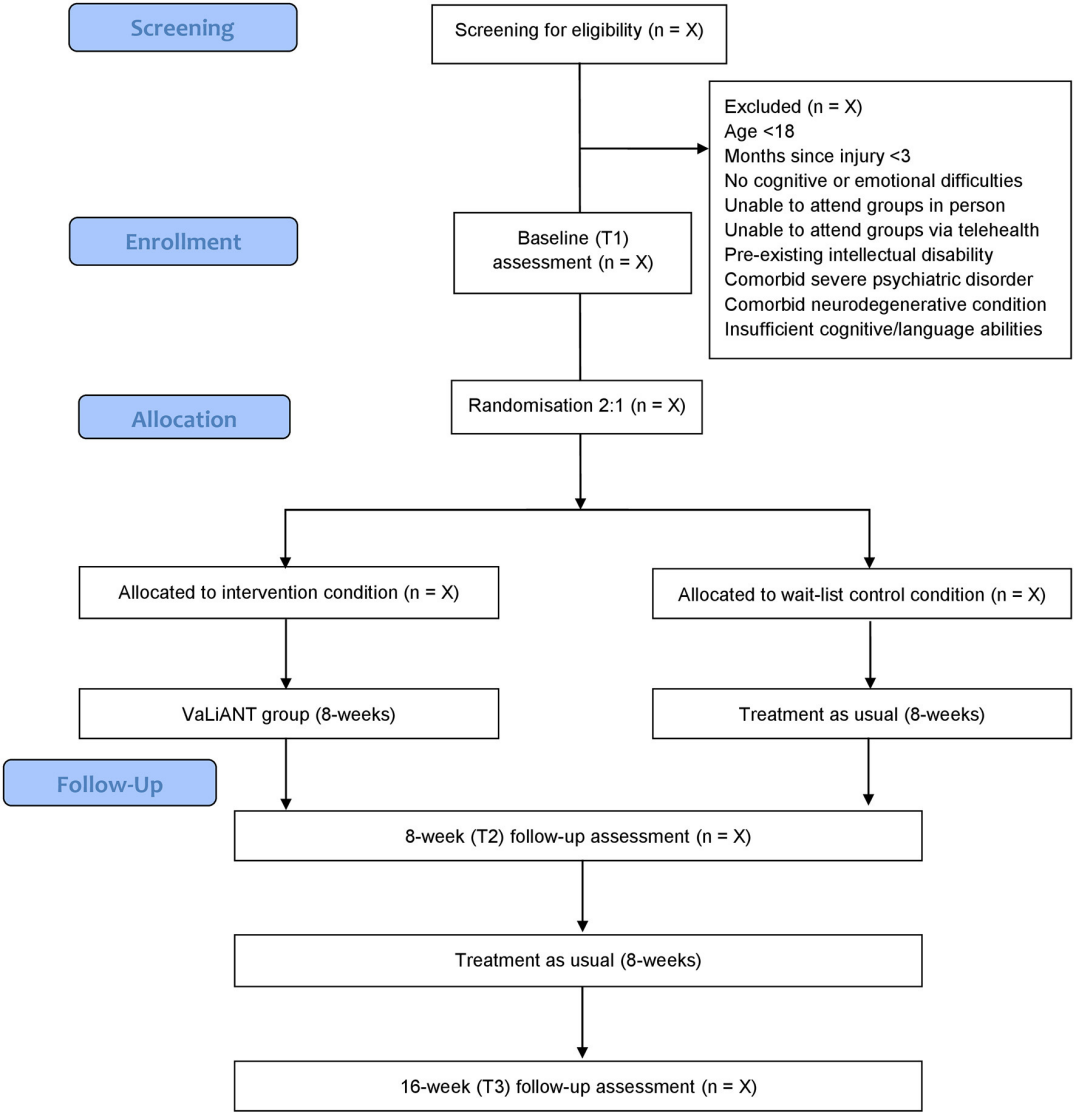
Study flow diagram. VaLiANT, Valued Living After Neurological Trauma.

This Phase II pilot study is a prospectively registered single center, two-arm, assessor-blinded, parallel groups RCT, comparing outcomes of the 8-week VaLiANT group intervention with treatment-as-usual waitlist control. Outcome measures are collected at baseline (T1), at an 8-week follow-up (T2), and at a 16-week follow-up from baseline (T3). An overview of the study procedure is summarized in Figure 1. This protocol was developed in accordance with the Standard Protocol Items: Recommendations for Interventional Trials (SPIRIT) guidelines (56). Methodological modifications made due to the impact of COVID-19 have been reported in line with SPIRIT Extension for RCTs Revised in Extenuating Circumstances (CONSERVE-SPIRIT) guidelines (57) throughout the text and summarized in a separate paragraph. The methodological quality of the trial will be evaluated using the Physiotherapy Evidence Database – Psycbite (PEDro-P) scale upon completion of the trial (58).

### Participants and Recruitment Process

This study is conducted at La Trobe Psychology Clinic (Melbourne, Australia); a psychology clinic at La Trobe University that also serves as a training clinic for postgraduate psychology students. Community-dwelling participants are identified either through self-referral or referral from a health professional. Recruitment methods include distribution of specific advertisement material (including flyers and weblinks) through local email listservs for clinicians/researchers who work with ABI (e.g., NPinOz, BRAINSPaN), local health services, practitioner networks, the Australian Stroke Clinical Registry (AuSCR), and relevant online portals for individuals living with ABI such as EnableMe (Stroke Foundation). Participants are required to be at least 3-months post-ABI (including stroke, TBI, brain tumor, hypoxic brain injury, and multiple sclerosis) before enrolment in the study; be 18 years of age or over; have reported cognitive and/or emotional difficulties (identified by self, close other and/or clinician in initial screening); and be able to attend the group program in person at La Trobe University Psychology Clinic or *via* telehealth during periods of COVID-19 related restriction. Exclusion criteria include pre-existing intellectual disability, severe psychiatric disorder, comorbid neurodegenerative condition, and insufficient cognitive and/or language abilities to complete outcome measures or participate in the intervention. Participant eligibility is determined *via* telephone screening conducted by the project coordinator (a trainee clinical neuropsychologist) prior to enrolment into the study.

### Intervention

The VaLiANT program is a manualized group intervention that concurrently targets cognition and emotion by integrating cognitive rehabilitation and ACT techniques to improve engagement in valued activities following ABI. The program consists of eight 2-h group sessions delivered either in-person or *via* telehealth during periods of COVID-19 restrictions, with group sizes ranging from three to eight participants. The intervention was developed by the authors, drawing on their clinical and research expertise, however evidence-based ACT and cognitive rehabilitation techniques and materials were adapted from existing manualized treatments to supplement the new content (46, 59–62). Group delivery was chosen due to a number of factors: 1) the cost-effectiveness compared to individual treatment, 2) the additional benefit of group discussion and the sharing of ideas for particular topics e.g., strategies to manage particular difficulties following ABI, and 3) to address social isolation and provide access to other individuals with shared experience. A number of small revisions were made to the manual and treatment delivery following completion of the Phase I study (55): 1) additional scaffolding was added in Session 2 to assist participants with linking their values to behavior, 2) a mindfulness exercise was included in every session (previously was in most but not all sessions), and 3) email reminders for the homework activities were sent to participants at the end of each calendar week. The treatment manual and resources will be published following completion of the trial. For more information on the intervention content and additional modifications made due to COVID-19, please see Tables 1, 2.

**Table 1:** TIDieR checklist describing the Valued Living After Neurological Trauma intervention and telehealth modifications.

**Item**	**Telehealth modification**
*1. Name* Valued Living After Neurological Trauma (VaLiANT)	
*2. Why* Existing interventions that target cognition and mood separately have displayed variable effectiveness and limited generalizability to broader outcomes (e.g., participation and quality of life) which may relate to the lack of integration between cognition and emotional symptoms. Valued living has been associated with better functional and psychosocial outcomes and has been identified as a potential treatment target following brain injury. VaLiANT utilizes a combined therapeutic approach that targets both cognitive impairment and mood disturbance with an overall focus on improving valued living. This represents a novel approach to improving outcomes post brain-injury.	
*3. What (materials)* *Treatment manual:* Each therapist delivering the intervention has access to a treatment manual outlining the treatment objectives, content to be covered each week, participant handouts and materials, and homework activities. The manual provides detailed instructions on how to cover each treatment component or activity, including suggested wording or phrasing, and prompts for enhancing or directing discussions.	
*Participant worksheets and handouts:* Each week participants receive a hard-copy package of psychoeducational handouts, recordings of mindfulness activities, and worksheets that are completed during the session or between sessions.	During periods of telehealth delivery, these materials are provided electronically using either cloud-sharing or *via* email.
*PowerPoint slides:* Each session is supported by PowerPoint slides displayed on an electronic overhead projector. Participants receive printed copies of the PowerPoint slides with space to take written notes during sessions.	During periods of telehealth delivery these are provided electronically using either cloud-sharing or *via* email.
*Values cards:* Hard-copy values cards specifically designed for the intervention are provided to participants within session for values card-sorting activities.	During periods of telehealth delivery, the values cards and associated activities are accessed *via* a custom-made electronic application hosted on a cloud platform (http://www.heroku.com).
*Sultanas:* Sultanas are provided to participants within session for a mindful eating exercise in Session 3.	Participants are instructed to bring a dried fruit or similar substitute to the relevant session. This is included as part of their homework from the previous week and an email reminder is sent prior to the session.
*Materials for passengers on the bus exercise:* Post it notes and values cards are used for an *in-vivo* passengers on the bus exercise.	The materials are substituted for extra PowerPoint slides.
*Whiteboard:* A whiteboard is used within multiple sessions for group discussion and brainstorming activities.	The electronic whiteboard function on Zoom is used instead.
*Pens:* Participants are provided with pens to take written notes during sessions	Pens are not provided during telehealth delivery.
*Computer/tablet and internet:* Not applicable	Participants are required to have their own computer or tablet device with a webcam and stable internet connection.
*4. What (procedures)* Every week of the VaLiANT program focusses on a different value domain (e.g., health, work/productive activities, leisure, relationships). Each session begins with a review of the previous week's homework. Following this, participants explore and identify their important values in that week's value domain (*via* the card-sort activity) and select one value to focus on over the following week. Participants then generate SMART (specific, measurable, achievable, relevant to the value, and time-bound) goals or “committed actions” that are consistent with the chosen value and can be done over the coming week. This process is supported by the group facilitators. The remainder of each session focusses on facilitating implementation of committed actions. Psychoeducation and various activities are used to teach cognitive compensatory strategies and ACT techniques such as mindfulness, including in-session practice of those strategies. With further support from facilitators, participants identify potential cognitive or emotional barriers to their committed actions (e.g., forgetfulness or low motivation) and select appropriate strategies to enable valued living. Most activities involve group discussion to encourage reflection and exchange of ideas amongst participants. Weekly homework activities include completing the selected committed actions and other tasks that aim to increase implementation of taught strategies into everyday life. Further information on the content of each session can be found in Table 2 and the published outline of the treatment manual [(55); supplemental material].	
*5. Who Provided* The VaLiANT intervention is facilitated by a senior Clinical Neuropsychologist experienced in working with individuals with ABI and expertise in delivering group-based interventions, cognitive rehabilitation, and ACT. An additional two clinicians assist with facilitation of each group. These are primarily trainee psychologists assisting with the delivery of 1 – 2 groups as part of their postgraduate clinical neuropsychology or clinical psychology training. The assisting clinicians are provided with prior training and supervision by the senior facilitator including didactic instruction and observational learning by watching recordings of previous sessions. Quality of intervention delivery and group facilitation skills are monitored during each session by the senior facilitator, and feedback provided during supervision which occurs after every session.	
*6. How* The VaLiANT intervention is intended to be delivered in-person on a weekly basis in a group environment (ranging from 3 to 8 group members).	Following the onset of the COVID-19 pandemic, the intervention was redeveloped to be deliverable *via* telehealth using videoconferencing (Zoom).
*7. Where* In-person delivery of the intervention occurs at the La Trobe University Psychology Clinic (Melbourne, Australia).	Telehealth delivery of the intervention occurs in participants' homes, with facilitators either at the university or in their homes.
*8. When and How much* The intervention involves eight sessions that run weekly, for 2 h, over a period of 8–9 weeks (depending on breaks for public holidays).	
*9. Tailoring* The treatment manual is intended to be a flexible guide, whereby content can be tailored as long as key session objectives are met and key session components are delivered. For example, specific strategies for addressing cognitive and emotional barriers can be more strongly emphasized if several participants identify similar barriers (e.g., motivation) or only briefly covered if less relevant (e.g., word-finding strategies). There are other specific opportunities for tailoring of the intervention in particular sessions (e.g., additional “optional” activities to further explore core concepts) if the core content has been covered adequately with time remaining. This additional content is not required to cover the main concepts but allows some tailoring of the intervention depending on the abilities and preferences of group participants.	
*10. Modifications*	Telehealth adaptations included the development of an online program to present the values card sort task and associated weekly worksheet while also allowing facilitators to see what participants were doing in real time during these activities. This was essential to allow facilitators to support participants in generating committed actions in line with their chosen values. Due to the likelihood of technical difficulties and participants requiring additional assistance with the online tasks, the time allocated to some activities (e.g., identification of barriers) was reduced to allow more time for core components (e.g., strategies) to ensure that the key concepts were covered. Some strategies that are potentially not relevant for every participant (e.g., activity scheduling) were moved to “optional” discussions that are only covered if participants identify particular problems. Some activities were also modified slightly to allow for completion online e.g., a group experiential ACT exercise (“Passengers on the Bus”) which involves participants moving around the room became more discussion based.
*11. How well (planned)* All VaLiANT sessions are video recorded. To measure treatment fidelity, a random selection of at least 10% of the video-recorded intervention sessions will be assessed by an independent researcher trained in cognitive rehabilitation, Acceptance and Commitment Therapy and group interventions. They will evaluate whether clinicians were able to meet the session objectives and cover the prescribed content using a checklist based on the manual for each session (i.e., treatment adherence), as well as the clinicians' competence in group facilitation (i.e., therapist competence).	
Competency in group facilitation skills is assessed using the eNACT group facilitation competency checklist, a 4-point likert scale which measures the quality of the therapist's group facilitation across 16 skills from 0 = “skill not observed despite opportunity,” to 3 = “observed – done well” (63). An additional item was added to the checklist to assess whether facilitators had delivered the intervention in an ACT-consistent manner (“Therapist demonstrates psychological flexibility in interactions with participants i.e., shows openness, flexible self-awareness and engages in their own valued actions, even when difficult topics arise in the group”).	

*Item 12 [how well (actual): if intervention adherence or fidelity was assessed, describe the extent to which the intervention was delivered as planned] cannot be fully described until study completion and has been omitted*.

**Table 2:** Overview of Valued Living after Neurological Trauma (VaLiANT).

**Session**	**Content**
1	**Introduction to the program** Overview of the program, intervention aims, and main components Establishment of group rules and group facilitator role Getting to know each other and sharing of stories Introduction to values, valued living Values card sort exercise Passengers on the bus exercise Mindfulness breathing exercise Introduction to committed actions and experiential avoidance Homework– self-monitoring form and name association task
2	**Being Healthy—Sleep and fatigue management** Introduction to “being healthy” module Values card sort exercise Discussion on four pillars of health Sleep and fatigue psychoeducation and strategies Mindfulness body scan exercise Experiential avoidance discussion (optional) Identification of committed actions and barriers Introduction to S.M.A.R.T goals Introduction to and completion of the “way to valued living worksheet” Homework—rest break scheduling and completing committed actions
3	**Being Healthy – Diet and exercise management** Review of values selected in previous session Way to valued living worksheet Diet and exercise psychoeducation Exploration of barriers Passengers on the bus exercise Mindful eating exercise Identification of committed actions Strategies for planning, memory, pacing, and motivation Activity scheduling exercise Homework—mindful eating and completing committed actions
4	**Having a Purpose—Work, study, or participation in the community** Overview to “having a purpose” module Values card sort exercise Identification of committed actions Identification of barriers Mindfulness self-compassion exercise Strategies for prospective memory and completing complex tasks Homework—prospective memory task and completing committed actions
5	**Having a Purpose—Leisure activities** Introduction to leisure exercise Psychoeducation on mood and the importance of leisure Values card sort task Exploration of leisure activities Identification of committed actions Mindfulness of the senses exercise Barriers to leisure exploration & associated strategies Homework—leisure activity schedule and completing committed actions
6	**Connecting with Others—Relationships part I** Overview of “relationships module” Values card sort task Identification of strengths in relationships Identification of committed actions Barriers exploration Mindfulness S.T.O.P exercise Strategies for cognitive communication difficulties Homework—planning a difficult conversation and completing committed actions
7	**Connecting with Others—Relationships part II (friends/family session)** *Friends/family members (1st h)* Introduction to VaLiANT Introduction to values and valued living Values card sort exercise Introduction to barriers and communication changes following brain injury Managing difficult emotions exercise *Participants (1st h)* Reflection on relationships and values they would like to bring Identification of committed actions Addressing social barriers Passengers on the bus exercise *All together (2nd h)* Mindfulness S.T.O.P exercise Strategies to support communication of abilities and needs Open communication discussion Homework – have an open conversation and completing committed actions
8	**Review and future directions—Tying it all together** Review of values, committed actions, strengths, and barriers identified in previous sessions Re-identification of helpful strategies from previous sessions Mindfulness S.T.O.P exercise Post-traumatic growth discussion (optional) Future support options Conclusion

*Each session begins with a review of the previous session's content and homework tasks (excluding Session 1). In session 7 participants have the ability to bring a family member or friend who complete separate activities for the 1st h, before joining participants to practice communication strategies in the 2nd h*.

### Measures

The Warwick-Edinburgh Mental Wellbeing Scale (WEMWBS) was selected as the primary outcome measure as it captures the broader adjustment and quality of life outcomes that VaLiANT targets, and the majority of participants displayed reliable and clinically significant improvements on the measure during the Phase I evaluation of VaLiANT (55). The WEMWBS is a 14-item questionnaire that measures the frequency of positive mental health and wellbeing over the previous 2 weeks (64). Items such as “I've been feeling optimistic about the future” are rated on a 5-point Likert scale with higher scores indicating greater wellbeing (total score range 14–70). The scale demonstrates good internal consistency (0.91), test-retest reliability (0.83), and criterion validity (64) and has been used in a previous ABI RCT (51).

The Valued Living Questionnaire – Comprehension Support version (VLQ-CS^1^) was developed by members of the research team as an adaptation of the original VLQ, following evidence that multiple comprehension errors were made by people with ABI on the original measure^2^. The VLQ-CS is designed to suit to the needs of individuals with cognitive and/or communication difficulties, and includes visual communication supports, simplified instructions and examples of value-consistent behaviors to aid understanding. Ten value domains (e.g., family, work) are rated for importance on a 10-point scale (higher scores = higher importance). For domains with an importance rating ≥5, the extent to which time spent on value-consistent behaviors in that domain over the last week was “ideal” is then rated on a 10-point “consistency” scale (higher scores = more ideal). A composite score is derived by calculating the mean of the products of the importance and consistency scores. The VLQ-CS has been validated for use with ABI with greater test-retest reliability than the original measure ^1^.

Mood is assessed with the Hospital Anxiety and Depression Scale [HADS; (65)] and the Depression Anxiety Stress Scales [DASS-21; (66)]. The inclusion of both measures was based on previous research in brain injury which indicated that the HADS-A is more sensitive to clinically relevant symptoms of anxiety while the DASS-D is more sensitive to clinically relevant symptoms of depression (67).

All other sample characterization measures and secondary outcome measures are summarized in Table 3.

**Table 3:** Timing of outcome measures.

**Outcome domain**	**Measure**	**T1**	**T2**	**T3**
**Sample characterization**
Premorbid intellectual ability	Test of Premorbid Functioning (68)	X		
Verbal memory	Rey Auditory Verbal Learning Test (69)	X		
Cognitive flexibility^*^	Trail Making Test—written (70) and oral (71) versions	X		
Idea generation	Controlled Oral Word Association Test (72)	X		
Treatment expectancy	The Credibility/Expectancy Questionnaire (73)	X		
**Primary outcome**
Wellbeing	The Warwick-Edinburgh Mental Wellbeing Scale (64)	X	X	X
**Secondary outcomes**
Mood	Hospital Anxiety and Depression Scale (65)	X	X	X
	Depression Anxiety Stress Scales – 21 (66)	X	X	X
Valued living^**^	Valued Living Questionnaire – original (74) and comprehension support^1^ version	X	X	X
	Valuing Questionnaire (44)	X	X	X
Psychological flexibility	The Acceptance and Action Questionnaire after brain injury (75)	X	X	X
Quality of life^**^	World Health Organization Quality of Life scale (76)	X	X	X
Psychological adjustment^**^	The Head Injury Semantic Differential Scale – III (77)	X	X	X
Community participation^**^	The Community Integration Questionnaire – original (78) and revised (79) versions	X	X	X
Post-traumatic growth	The Changes in Outlook Questionnaire – Short form (80)	X	X	X
Cognitive strategy use	Self-report strategy use checklist (81)	X	X	X
Subjective memory functioning	The Everyday Memory Questionnaire – Revised (82)	X	X	X
Self-Efficacy	The TBI Self-Efficacy Scale (83)	X	X	X

*T1, baseline assessment; T2, 8-week follow-up assessment; T3, 16-week follow-up assessment*.

* *Indicates measures that were adapted to be deliverable via telehealth*.

** *Indicates measures that were included or adapted following trial commencement*.

### Feasibility and Acceptability Measures

Feasibility of the trial design will be assessed against the following criteria: 1) recruitment of the minimum number of participants required to run quarterly groups throughout the study period (minimum of 3 participants per group); 2) acceptable participant drop-out rates in intervention and control conditions (<20%); 3) adequate outcome assessment completion rates (≥80%); and 4) successful blinding of outcome assessors (≥90%). Consistent with the Phase I study (55), feasibility of the intervention will be assessed against: 1) group attendance rates (≥80% overall participant attendance); 2) and homework completion rates (≥50% completion rate for participants in attendance for each session). Acceptability of the intervention is measured by asking participants to rate their level of confidence in recommending the VaLiANT program to a friend who experiences similar problems (1 = “Not at all confident,” 9 = “Very confident”). The intervention will be deemed “acceptable” if the mean rating is ≥80% (i.e., ≥7.2/9).

### Randomization and Blinding

Randomization is performed by a researcher independent from the study using an online generator known as Research Randomizer (https://www.randomizer.org). Eligible participants are randomly assigned to the intervention condition or control condition with an allocation ratio of 2:1 (Intervention: Control). This allocation ratio was selected to optimize recruitment rates and maximize the number of people experiencing the intervention to allow for exploration of treatment dimensions and predictors of outcome (84, 85). Randomly permuted block sizes of 3, 6, or 9 are used to ensure a balanced allocation ratio. Group allocation is concealed, either in sequentially numbered sealed opaque envelopes (pre-COVID) or electronically *via* sequentially numbered word-documents uploaded to a protected cloud-sharing platform (post-COVID), which are opened at the end of the baseline (T1) assessment. The outcome assessors at T2 and T3 are research assistants blinded to condition allocation. Participants are reminded to not disclose their allocation during assessments, and all instances of unblinding are recorded. If unblinding occurs during a T2 assessment, then a different blinded research assistant conducts that participant's T3 assessment.

### Procedure

The VaLiANT group is planned to run quarterly with an associated participant intake period prior to commencement of each group. Potential participants undergo screening to ensure eligibility before informed consent is obtained. For each intake, all eligible participants attend an initial baseline assessment (T1) which includes all baseline sample characterization measures and primary and secondary outcome measures. Randomization occurs immediately following the T1 assessment. In addition to their usual care, participants in the treatment condition then attend the 8-week VaLiANT group program at the La Trobe University Psychology Clinic, or *via* telehealth (Zoom videoconferencing) during periods of COVID-19 restrictions, while control participants undergo treatment-as-usual (i.e., their usual care). Participation in other treatment during the trial is documented, including the frequency and type of treatment. VaLiANT group sessions are facilitated by an experienced clinical neuropsychologist with assistance from two trainee psychologists. All sessions are video-recorded. Outcome assessments occur within 1–2 weeks following the intervention/waiting period (T2), and at an 8-week follow-up (T3). All assessments take roughly 90 min and are administered by assessors blinded to condition allocation. Assessments are conducted at the La Trobe University Psychology Clinic or in participants' homes if preferable. During periods where COVID-19 restrictions apply, assessments are conducted over Zoom videoconferencing.

### Data Management

During the trial, hard copy information is stored at La Trobe Psychology Clinic in a locked cabinet while electronic information is stored on secure electronic databases, accessible only by the project coordinator, chief investigator, and research assistants. Prior to data analysis, all values will be checked for plausibility. Data will be retained for 7 years after completion of the project and then destroyed by securely deleting electronic records (including video and audio recordings) and shredding all paper records.

### Sample Size Calculation

A power analysis was conducted using 5,000 simulations within the SimR package for R (86) to determine if the maximum possible sample size during the data collection period (*N* = 64) was sufficient for the statistical analyses. A previous evaluation of an ACT-based intervention following stroke reached a moderate group-by-time effect (η2 = 0.07) on the Warwick and Edinburgh Mental Wellbeing Scale (51). Accordingly, a minimum sample of 52 participants is required to achieve statistical power for a linear mixed-effect model with a 2 (group) x 3 (time) design (80% power, α = 0.05). Allowing for an attrition rate of 10%, an N of 58 is adequate to perform the primary analyses.

### Statistical Analysis

Main analyses will follow an intention-to-treat approach. Little's missing completely at random (MCAR) analysis will be conducted to determine if data are MCAR (87). If <5% of data is MCAR, the appropriate data imputation technique will be employed to deal with missing values [likely Markov chain Monte Carlo method; (88)]. Univariate outliers (z +/− 1.96 SD) will be adjusted using a winsorising solution (89). Univariate checks of normality (skewness > +/− 2.58 SD) will be conducted, and variables that violate the criterion will be corrected to normal using appropriate data transformation (90). Primary and secondary outcomes will be analyzed with linear mixed models, with fixed effects of time and group, and participants modeled as random effects. The estimated marginal means from the model will be used to calculate effect sizes (Cohen's *d*) to illustrate change in both between group and timepoint contrasts. The results of the fixed effects estimates for the main effects and interaction terms will be presented as standardized *B* values and all analyses will use a two-sided alpha level of 0.05. These analyses will be conducted using JASP (91). Finally, as an adjunct to the linear mixed models, the Crawford and Howell measure of reliable change (92), which is suitable for serial testing, will be calculated for the primary outcome (93). The proportion of participants achieving reliable change in each group at both time-points will then be compared with 2 × 2 Chi square tests of independence.

### Protocol Amendments Due to COVID-19

The COVID-19 pandemic has resulted in a number of essential methodological changes to the original study protocol. The trial commenced in August 2019, and then in March 2020 it was paused for 6-months after the onset of the pandemic, given that restrictions prevented in-person assessments and intervention delivery. To allow the trial to continue, the research team redeveloped the study protocol for telehealth delivery on Zoom. To allow for outcome assessments to be conducted *via* telehealth, data collection measures have been moved from Qualtrics to REDCap, the randomization schedule has shifted from opaque envelopes to sequentially numbered word-documents uploaded to a cloud-sharing platform, and the paper-and-pencil Trail Making Test has been substituted with the oral version for telehealth baseline assessments. A telehealth version of the VaLiANT intervention was also developed (see Table 1). For analyses, telehealth delivery will be treated as a substitution for in-person delivery rather than as a separate treatment arm. In-person delivery remains the preferred modality and will be utilized where possible. Changes have been made to the inclusion criteria such that participants are required to be able to attend assessments and the intervention both in-person and *via* telehealth, to allow flexibility with changing restrictions.

In addition, a number of other non-essential modifications have been made following the opportunity to reflect on the trial design during the trial's pause in 2020, and further evidence accumulated during that period. In weighing up whether to introduce these changes after trial commencement, the research team considered the fact that this is a feasibility Phase II trial and therefore opted to make changes to optimize trial design and better inform a future Phase III trial. Initially, randomization occurred in randomly permuted block sizes of 6. However, it was possible for the baseline assessor to deduce the final participant's allocation in one block based on previous allocations. As such, varying block sizes (3, 6, or 9) were introduced to maximize blinding of the baseline assessor in future assessments. The Community Integration Questionnaire was updated to the revised version which includes an additional electronic social networking scale, relevant in the context of social distancing requirements. The 26-item World Health Organization Quality of Life scale (WHOQOL-BREF) and the Head Injury Semantic Differential Scale – III (HISDS-III) were included as additional outcome measures to provide more comprehensive measurement of quality of life and psychological adjustment. Finally, the Valued Living Questionnaire (VLQ) was replaced with an adapted version (VLQ-CS) following identification of validity issues with the original measures due to frequent comprehension errors made by those with ABI^2^. All modifications occurred during the pause in data collection (March – September 2020) with the exception of changes to the Valued Living Questionnaire which occurred in September 2019.

## Discussion

There is a recognized need for trials evaluating complex, multi-domain, person-centered interventions post-ABI that aim to improve rehabilitation outcomes beyond injury-related impairments (e.g., cognitive and mood changes) by also targeting overall adjustment to injury, meaningful participation, and quality of life (33). While a number of complex interventions have integrated cognitive rehabilitation and psychological therapy with subsequent positive long-term effects, these interventions are lengthy and require high treatment dosage which limits their implementation into routine practice. The proposed RCT aims to build on our Phase I findings (55) by evaluating the efficacy, feasibility, and acceptability of the 8-week VaLiANT group program against a treatment-as-usual waitlist control.

The study has several strengths. Many aspects of the current trial design were piloted and found to be feasible in the previous Phase I study (e.g., recruitment rates, outcome assessment completion rates). The inclusion criteria for the study are broad and include multiple forms of ABI in comparison to many intervention studies which focus on a single cohort (e.g., stroke). It is therefore anticipated that the sample will be fairly heterogeneous, supporting generalization of the study findings to the broader ABI community and implementation into ABI rehabilitation services (which are rarely devoted to a single cohort), while potentially also allowing for greater exploration of predictors of treatment outcome depending on the sample size. Additionally, the intervention was developed by a multi-disciplinary team based on current evidence (including existing manualized treatment approaches). The intervention includes specific adaptations to meet the needs of those with ABI, it can be delivered both in-person and *via* telehealth aiding flexibility, and it is group-based which may be more cost-effective than individual therapy.

Several limitations are also acknowledged. The study has been conducted during the COVID-19 pandemic in Melbourne, Australia which has been subject to multiple extended and rolling lockdowns throughout the study period. Study outcomes may be impacted during periods of restriction due to limited opportunities for intervention-related behavior change and the overall negative impact on mood and wellbeing. Additionally, there may be rapid improvements in both study conditions when lockdowns or restrictions are eased. The pandemic has also necessitated a number of changes to the study design and methodology. In particular, the variable modality of intervention delivery between participants (i.e., in-person, telehealth, or blended) may impact intervention outcome. The associated change in inclusion criteria, which requires participants to have both in-person and telehealth capacity, may also lead to a restricted sample by limiting the intervention to higher-functioning individuals. The study is also limited to English speaking individuals with sufficient cognitive and language capacity to complete the outcome assessments and participate in the group intervention, which may further limit the generalizability of findings, particularly to those with significant aphasia.

This study will extend current knowledge on the utility of complex interventions and will add to the growing body of evidence investigating the role of valued living as an important treatment target following ABI. The study findings will also add to recent evidence supporting the adaptation of ACT for those with ABI. Given that previous investigations have focused on purely ACT-based interventions without a cognitive rehabilitation component, this study will demonstrate the utility of incorporating ACT principles within a more holistic intervention framework. Finally, study findings will help determine the feasibility and implementation of a definitive Phase III RCT.

## Data Availability Statement

The original contributions presented in the study are included in the article/supplementary material, further inquiries can be directed to the corresponding author.

## Ethics Statement

The studies involving human participants were reviewed and approved by La Trobe University Human Research Ethics Committee. The patients/participants provided their written informed consent to participate in this study.

## Author Contributions

NS, EM, DG, LK, BD-B, RN, and DW were involved in the development of the intervention and the study protocol. BW was responsible for the statistical analysis plan. NS, DW, and BD-B are responsible for project coordination and administration. NS and DW were responsible for writing and revising the manuscript. All authors have read and approved the final version of the manuscript.

## Funding

This work was supported by the La Trobe University Sport, Exercise, and Rehabilitation Research Focus Area Grant Ready Scheme. The funding body has no role in the study design, data collection, data analysis and interpretation, or write-up of the manuscript.

## Conflict of Interest

The authors declare that the research was conducted in the absence of any commercial or financial relationships that could be construed as a potential conflict of interest.

## Publisher's Note

All claims expressed in this article are solely those of the authors and do not necessarily represent those of their affiliated organizations, or those of the publisher, the editors and the reviewers. Any product that may be evaluated in this article, or claim that may be made by its manufacturer, is not guaranteed or endorsed by the publisher.

## Footnotes

^1^Wong D, Miller H, Lawson D, Borschmann K, Sathananthan N, Kamberis N, et al. *Development and validation of the Valued Living Questionnaire - Comprehension Support Version* (in preparation).

^2^Miller H, Lawson D, Power E, das Nair R, Sathananthan N, Wong D. *How do people with acquired brain injury interpret the Valued Living Questionnaire? A cognitive interviewing study* (under review).

## References

[1] Rabinowitz AR Levin HS. Cognitive sequelae of traumatic brain injury. Psychiatric Clin North Am. (2014) 37:1–11. 10.1016/j.psc.2013.11.004 24529420 PMC3927143

[2] Mellon L Brewer L Hall P Horgan F Williams D Hickey A. Cognitive impairment six months after ischaemic stroke: a profile from the ASPIRE-S study. BMC Neurol. (2015) 15:31. 10.1186/s12883-015-0288-2 25879880 PMC4359388

[3] Hackett ML Pickles K. Part I: frequency of depression after stroke: an updated systematic review and meta-analysis of observational studies. Int J Stroke. (2014) 9:1017–25. 10.1111/ijs.12357 25117911

[4] Campbell Burton CA Murray J Holmes J Astin F Greenwood D Knapp P. Frequency of anxiety after stroke: a systematic review and meta-analysis of observational studies. Int J Stroke. (2013) 8:545–59. 10.1111/j.1747-4949.2012.00906.x 23013268

[5] Anson K Ponsford J. Coping and emotional adjustment following traumatic brain injury. J Head Trauma Rehabilitation. (2006) 21:248–59. 10.1097/00001199-200605000-00005 16717502

[6] Madsen T Erlangsen A Orlovska S Mofaddy R Nordentoft M Benros ME. Association between traumatic brain injury and risk of suicide. JAMA. (2018) 2018:320. 10.1001/jama.2018.10211 30120477 PMC6142987

[7] Ponsford J Downing MG Olver J Ponsford M Acher R Carty M. Longitudinal follow-up of patients with traumatic brain injury: Outcome at two, five, and ten years post-injury. J Neurotrauma. (2014) 31:64–77. 10.1089/neu.2013.2997 23889321

[8] Nijsse B van Heugten CM van Mierlo ML Post MWM de Kort PLM Visser-Meily JMA. Psychological factors are associated with subjective cognitive complaints 2 months post-stroke. Neuropsychol Rehabilit. (2017) 27:99–115. 10.1080/09602011.2015.1065280 26207868

[9] Kimonides S Cavuoto MG De Silva L Kinsella GJ. The role of subjective cognitive complaints and depressive symptoms in social re-integration following stroke: a mediation explanation in a cross-sectional sample. Topics Stroke Rehabilit. (2018) 25:514–20. 10.1080/10749357.2018.1489570 30040053

[10] Pickelsimer EE Selassie AW Sample PL Heinemann A Gu JK Veldheer LC. Unmet service needs of persons with traumatic brain injury. J Head Trauma Rehabilit. (2007) 22:1–13. 10.1097/00001199-200701000-00001 17235226

[11] Andrew NE Kilkenny M Naylor R Purvis T Lalor E Moloczij N. Understanding long-term unmet needs in Australian survivors of stroke. Int J Stroke. (2014) 9:106–12. 10.1111/ijs.12325 25042019

[12] Gadidi V Katz-Leurer M Carmeli E Bornstein NM. Long-term outcome poststroke: predictors of activity limitation and participation restriction. Arch Phys Med Rehabilitation. (2011) 92:1802–8. 10.1016/j.apmr.2011.06.014 22032214

[13] Stolwyk RJ Mihaljcic T Wong DK Chapman JE Rogers JM. Post-stroke cognitive impairment negatively impacts activity and participation outcomes: a systematic review and meta-analysis. Stroke. (2021) 52:748–60. 10.1161/STROKEAHA.120.032215 33493048

[14] Mole JA Demeyere N. The relationship between early post-stroke cognition and longer term activities and participation: A systematic review. Neuropsychol Rehabilit. (2020) 30:346–70. 10.1080/09602011.2018.1464934 29712538

[15] Theadom A Starkey N Barker-Collo S Jones K Ameratunga S Feigin V. Population-based cohort study of the impacts of mild traumatic brain injury in adults four years post-injury. PLoS ONE. (2018) 13:e0191655. 10.1371/journal.pone.0191655 29385179 PMC5791998

[16] Barker-Collo S Feigin VL Parag V Lawes CM Senior H. Auckland stroke outcomes study. Part 2: Cognition and functional outcomes 5 years poststroke. Neurology. (2010) 75:1608–16. 10.1212/WNL.0b013e3181fb44c8 21041784

[17] Wood RL Rutterford NA. Demographic and cognitive predictors of long-term psychosocial outcome following traumatic brain injury. J Int Neuropsychol Soc. (2006) 12:350–8. 10.1017/S1355617706060498 16903127

[18] Feigin VL Barker-Collo S Parag V Senior H Lawes CM Ratnasabapathy Y. Auckland Stroke Outcomes Study. Part 1: Gender, stroke types, ethnicity, and functional outcomes 5 years poststroke. Neurology. (2010) 75:1597–607. 10.1212/WNL.0b013e3181fb44b3 21041783

[19] Tse T Linden T Churilov L Davis S Donnan G Carey LM. Longitudinal changes in activity participation in the first year post-stroke and association with depressive symptoms. Disabillity Rehabilit. (2018) 2018:1–8. 10.1080/09638288.2018.1471742 29889570

[20] Draper K Ponsford J Schonberger M. Psychosocial and emotional outcomes 10 years following traumatic brain injury. J Head Trauma Rehabilit. (2007) 22:278–87. 10.1097/01.HTR.0000290972.63753.a7 17878769

[21] Kutlubaev MA Hackett ML. Part II: predictors of depression after stroke and impact of depression on stroke outcome: an updated systematic review of observational studies. Int J Stroke. (2014) 9:1026–36. 10.1111/ijs.12356 25156411

[22] Rastenyte D Kranciukaite D. Poststroke depression and its impact on quality of life. Medicina. (2007) 43:1–9. 10.3390/medicina43010001 17297278

[23] De Wit L Theuns P Dejaeger E Devos S Gantenbein AR Kerckhofs E. Long-term impact of stroke on patients' health-related quality of life. Disabillity Rehabilit. (2017) 39:1435–40. 10.1080/09638288.2016.1200676 27385479

[24] World Health Organization. International Classification of Functioning, Disability and Health: ICF. Geneva: World Health Organization (2001).

[25] Elliott M Parente F. Efficacy of memory rehabilitation therapy: a meta-analysis of TBI and stroke cognitive rehabilitation literature. Brain Injury. (2014) 28:1610–6. 10.3109/02699052.2014.934921 25058353

[26] Velikonja D Tate R Ponsford J McIntyre A Janzen S Bayley M. INCOG recommendations for management of cognition following traumatic brain injury, part V: memory. J Head Trauma Rehabilit. (2014) 29:369–86. 10.1097/HTR.0000000000000069 24984098

[27] das Nair R Cogger H Worthington E Lincoln NB. Cognitive rehabilitation for memory deficits after stroke. Cochrane Database Syst Rev. (2016) 9:CD002293. 10.1002/14651858.CD002293.pub3 27581994 PMC6457594

[28] Withiel TD Wong D Ponsford JL Cadilhac DA New P Mihaljcic T. Comparing memory group training and computerized cognitive training for improving memory function following stroke: A phase II randomized controlled trial. J Rehabilit Med. (2019) 51:343–51. 10.2340/16501977-2540 30815708

[29] Loetscher T Potter KJ Wong D das Nair R. Cognitive rehabilitation for attention deficits following stroke. Cochrane Database Syst Rev. (2019) 2019:CD002842. 10.1002/14651858.CD002842.pub3 31706263 PMC6953353

[30] Wang SB Wang YY Zhang QE Wu SL Ng CH Ungvari GS. Cognitive behavioral therapy for post-stroke depression: A meta-analysis. J Affective Disord. (2018) 235:589–96. 10.1016/j.jad.2018.04.011 29704854

[31] Gertler P Tate RL Cameron ID. Non-pharmacological interventions for depression in adults and children with traumatic brain injury. Cochrane Database Syst Rev. (2015) 2015:CD009871. 10.1002/14651858.CD009871.pub2 26663136 PMC8761477

[32] Ponsford J Lee NK Wong D McKay A Haines K Alway Y. Efficacy of motivational interviewing and cognitive behavioral therapy for anxiety and depression symptoms following traumatic brain injury. Psychol Med. (2016) 46:1079–90. 10.1017/S0033291715002640 26708017

[33] Doering B Exner C. Combining neuropsychological and cognitive-behavioral approaches for treating psychological sequelae of acquired brain injury. Curr Opin Psychiatry. (2011) 24:156–61. 10.1097/YCO.0b013e328343804e 21206272

[34] Wright CJ Zeeman H Biezaitis V. Holistic practice in traumatic brain injury rehabilitation: Perspectives of health practitioners. PLoS ONE. (2016) 11:e0156826. 10.1371/journal.pone.0156826 27270604 PMC4894634

[35] Shany-Ur T Bloch A Salomon-Shushan T Bar-Lev N Sharoni L Hoofien D. Efficacy of postacute neuropsychological rehabilitation for patients with acquired brain injuries is maintained in the long-term. J Int Neuropsychol Soc. (2020) 26:130–41. 10.1017/S1355617719001024 31983377

[36] Holleman M Vink M Nijland R Schmand B. Effects of intensive neuropsychological rehabilitation for acquired brain injury. Neuropsychol Rehabilit. (2018) 28:649–62. 10.1080/09602011.2016.1210013 27487525

[37] Cicerone KD Mott T Azulay J Sharlow-Galella MA Ellmo WJ Paradise S. A randomized controlled trial of holistic neuropsychologic rehabilitation after traumatic brain injury. Arch Phys Med Rehabilit. (2008) 89:2239–49. 10.1016/j.apmr.2008.06.017 19061735

[38] Exner C Doering BK Conrad N Künemund A Zwick S Kühl K. Integrated neuropsychological and cognitive behavioural therapy after acquired brain injury: A pragmatic randomized clinical trial. Neuropsychol Rehabilit. (2021) 2021:1–35. 10.1080/09602011.2021.1908902 33818305

[39] Domensino A-F Verberne D Prince L Fish J Winegardner J Bateman A. Client experiences with holistic neuropsychological rehabilitation: “It is an ongoing process”. Neuropsychol Rehabilit. (2021) 2021:1–23. 10.1080/09602011.2021.1976222 34596002

[40] Pais C Ponsford JL Gould KR Wong D. Role of valued living and associations with functional outcome following traumatic brain injury. Neuropsychol Rehabilit. (2019) 29:625–37. 10.1080/09602011.2017.1313745 28421872

[41] Baseotto MC Morris PG Gillespie DC Trevethan CT. Post-traumatic growth and value-directed living after acquired brain injury. Neuropsychol Rehabilit. (2020) 2020:1–20. 10.1080/09602011.2020.1798254 32715913

[42] Graham CD Gouick J Krahe C Gillanders D. A systematic review of the use of Acceptance and Commitment Therapy (ACT) in chronic disease and long-term conditions. Clin Psychol Rev. (2016) 46:46–58. 10.1016/j.cpr.2016.04.009 27176925

[43] Sheppard SC Forsyth JP Hickling EJ Bianchi J. A novel application of acceptance and commitment therapy for psychosocial problems associated with multiple sclerosis: Results from a half-day workshop intervention. Int J MS Care. (2010) 12:200–6. 10.7224/1537-2073-12.4.200

[44] Smout M Davies M Burns N Christie A. Development of the Valuing Questionnaire (VQ). J Context Behav Sci. (2014) 3:164–72. 10.1016/j.jcbs.2014.06.001

[45] Van Bost G Van Damme S Crombez G. The role of acceptance and values in quality of life in patients with an acquired brain injury: a questionnaire study. PeerJ. (2017) 5:e3545. 10.7717/peerj.3545 28695071 PMC5501966

[46] Whiting DL Deane F McLeod H Ciarrochi J Simpson G. Can acceptance and commitment therapy facilitate psychological adjustment after a severe traumatic brain injury? A pilot randomized controlled trial. Neuropsychol Rehabilit. (2020) 30:1348–71. 10.1080/09602011.2019.1583582 30789059

[47] Whiting DL Deane FP Simpson GK Ciarrochi J McLeod HJ. Acceptance and Commitment Therapy delivered in a dyad after a severe traumatic brain injury: A feasibility study. Clin Psychol. (2018) 22:230–40. 10.1111/cp.12118

[48] Sander AM Clark AN Arciniegas DB Tran K Leon-Novelo L Ngan E. A randomized controlled trial of acceptance and commitment therapy for psychological distress among persons with traumatic brain injury. Neuropsychol Rehabilit. (2021) 31:1105–29. 10.1080/09602011.2020.1762670 32408846

[49] McCoy T Philp A Kates-McElrath K. Acceptance and Commitment Therapy: A systematic review of psychological adjustment and reduction of psychological distress following a traumatic brain injury in adults. J Addiction Psychiatry Mental Health. (2021) 1:1–10.

[50] Graham CD Gillanders D Stuart S Gouick J. An acceptance and commitment therapy (ACT)-based intervention for an adult experiencing post-stroke anxiety and medically unexplained symptoms. Clin Case Stud. (2015) 14:83–97. 10.1177/1534650114539386

[51] Majumdar S Morris R. Brief group-based acceptance and commitment therapy for stroke survivors. Br J Clin Psychol. (2019) 58:70–90. 10.1111/bjc.12198 29999185

[52] Large R Samuel V Morris R. A changed reality: Experience of an acceptance and commitment therapy (ACT) group after stroke. Neuropsychol Rehabilit. (2020) 30:1477–96. 10.1080/09602011.2019.1589531 30924741

[53] Gillanders S Gillanders D. An acceptance and commitment therapy intervention for a woman with secondary progressive multiple sclerosis and a history of childhood trauma. Neuro-Disability Psychother. (2014) 2:19–40.

[54] Hill G Hynd N Wheeler M Tarran-Jones A Carrabine H Evans S. Living well with neurological conditions: Evaluation of an ACT-informed group intervention for psychological adjustment in outpatients with neurological problems. Neuropsychologist. (2017) 3:58–63.

[55] Sathananthan N Dimech-Betancourt B Morris E Vicendese D Knox L Gillanders D. A single-case experimental evaluation of a new group-based intervention to enhance adjustment to life with acquired brain injury: VaLiANT (valued living after neurological trauma). Neuropsychol Rehabilit. (2021) 30:1348–71. 10.1080/09602011.2021.1971094 34433379

[56] Chan AW Tetzlaff JM Altman DG Laupacis A Gøtzsche PC KrleŽa-Jerić K. SPIRIT 2013 statement: defining standard protocol items for clinical trials. Ann Intern Med. (2013) 158:200–7. 10.7326/0003-4819-158-3-201302050-00583 23295957 PMC5114123

[57] Orkin AM Gill PJ Ghersi D Campbell L Sugarman J Emsley R. Guidelines for reporting trial protocols and completed trials modified due to the COVID-19 pandemic and other extenuating circumstances: the CONSERVE 2021 statement. JAMA. (2021) 326:257–65. 10.1001/jama.2021.9941 34152382

[58] Murray E Power E Togher L McCabe P Munro N Smith K. The reliability of methodological ratings for speechBITE using the PEDro-P scale. Int J Lang Commun Disord. (2013) 48:297–306. 10.1111/1460-6984.12007 23650886

[59] Radford K Say M Thayer Z Miller LA. Making the Most of Your Memory: An Everyday Memory Skills Program. Sydney: ASSBI Resources (2010).

[60] Withiel TD Stolwyk RJ Ponsford JL Cadilhac DA Wong D. Effectiveness of a manualised group training intervention for memory dysfunction following stroke: a series of single case studies. Disability Rehabilit. (2020) 42:3033–42. 10.1080/09638288.2019.1579260 30978122

[61] O'Donoghue EK Morris EMJ Oliver JE Johns LC. ACT for Psychosis Recovery: A Practical Manual for Group-Based Interventions Using Acceptance and Commitment Therapy. Oakland, CA: Context Press/New Harbinger Publications (2018).

[62] Brassington L Ferreira NB Yates S Fearn J Lanza P Kemp K. Better living with illness: A transdiagnostic acceptance and commitment therapy group intervention for chronic physical illness. J Context Behav Sci. (2016) 5:208–14. 10.1016/j.jcbs.2016.09.001

[63] Wong D Grace N Baker K McMahon G. Measuring clinical competencies in facilitating group-based rehabilitation interventions: development of a new competency checklist. Clin Rehabilit. (2019) 33:1079–87. 10.1177/0269215519831048 30806075

[64] Tennant R Hiller L Fishwick R Platt S Joseph S Weich S. The Warwick-Edinburgh Mental Well-being Scale (WEMWBS): development and UK validation. Health Q Life Outcomes. (2007) 5:63. 10.1186/1477-7525-5-63 18042300 PMC2222612

[65] Zigmond AS Snaith RP. The hospital anxiety and depression scale. Acta Psychiatr Scand. (1983) 67:361–70. 10.1111/j.1600-0447.1983.tb09716.x 6880820

[66] Lovibond SH Lovibond PF. Manual for the Depression Anxiety Stress Scales. Sydney: Psychology Foundation (1995).

[67] Dahm J Wong D Ponsford J. Validity of the Depression Anxiety Stress Scales in assessing depression and anxiety following traumatic brain injury. J Affect Disord. (2013) 151:392–6. 10.1016/j.jad.2013.06.011 23830002

[68] Pearson N. Advanced Clinical Solutions for WAIS-IV and WMS-IV: Administration and Scoring Manual. San Antonio, TX: The Psychological Corporation (2009).

[69] Schmidt M. Rey Auditory Verbal Learning Test: A Handbook. Los Angeles, CA: Western Psychological Services (1996).

[70] Tombaugh TN. Trail making test A and B: normative data stratified by age and education. Arch Clin Neuropsychol. (2004) 19:203–14. 10.1016/S0887-6177(03)00039-8 15010086

[71] Ricker JH Axelrod BN. Analysis of an oral paradigm for the trail making test. Assessment. (1994) 1:47–51. 10.1177/1073191194001001007 9463499

[72] Lezak MD. Neuropsychological Assessment. Oxford: Oxford University Press (1995).

[73] Devilly GJ Borkovec TD. Psychometric properties of the credibility/expectancy questionnaire. J Behav Ther Experi Psychiatry. (2000) 31:73–86. 10.1016/S0005-7916(00)00012-4 11132119

[74] Wilson KG Sandoz EK Kitchens J Roberts M. The Valued Living Questionnaire: defining and measuring valued action within a behavioral framework. Psychol Rec. (2010) 60:249–72. 10.1007/BF03395706

[75] Whiting DL Deane FP Ciarrochi J McLeod HJ Simpson GK. Validating measures of psychological flexibility in a population with acquired brain injury. Psychol Assessment. (2015) 27:415–23. 10.1037/pas0000050 25419644

[76] Skevington SM Lotfy M O'Connell KA. The World Health Organization's WHOQOL-BREF quality of life assessment: Psychometric properties and results of the international field trial. A Report from the WHOQOL Group. Q Life Res. (2004) 13:299–310. 10.1023/B:QURE.0000018486.91360.00 15085902

[77] Tyerman A Humphrey M. Changes in self-concept following severe head injury. Int J Rehabilit Res. (1984) 7:11–24. 10.1097/00004356-198403000-00002 6735545

[78] Willer B Rosenthal M Kreutzer JS Gordon WA Rempel R. Assessment of community integration following rehabilitation for traumatic brain injury. J Head Trauma Rehabilit. (1993) 8:75–87. 10.1097/00001199-199308020-00009

[79] Callaway L Winkler D Tippett A Migliorini C Herd N Willer B. The Community Integration Questionnaire-Revised (CIQ-R). Melbourne, VIC: Summer Foundation (2014). 10.1111/1440-1630.12284 27072343

[80] Joseph S Alex Linley P Shevlin M Goodfellow B Butler LD. Assessing positive and negative changes in the aftermath of adversity: a short form of the Changes in Outlook Questionnaire. J Loss Trauma. (2006) 11:85–99. 10.1080/15325020500358241

[81] Radford K Say M Thayer Z Miller L. Making the Most of Your Memory: An Everyday Memory Skills Program. Sydney, NSW: ASSBI Resources (2010).

[82] Royle J Lincoln NB. The everyday memory questionnaire - revised: development of a 13-item scale. Disability Rehabilit. (2008) 30:114–21. 10.1080/09638280701223876 17852284

[83] Huckans M Pavawalla S Demadura T Kolessar M Seelye A Roost N. A pilot study examining effects of group-based Cognitive Strategy Training treatment on self-reported cognitive problems, psychiatric symptoms, functioning, and compensatory strategy use in OIF/OEF combat veterans with persistent mild cognitive disorder and history of traumatic brain injury. J Rehabil Res Dev. (2010) 47:43–60. 10.1682/JRRD.2009.02.0019 20437326 PMC4755481

[84] Torgerson DJ Torgerson CJ. Designing Randomised Trials in Health, Education and the Social Sciences: An Introduction. Palgrave Macmillan (2008). 10.1057/9780230583993

[85] Hey SP Kimmelman J. The questionable use of unequal allocation in confirmatory trials. Neurology. (2014) 82:77–9. 10.1212/01.wnl.0000438226.10353.1c 24306005 PMC3873626

[86] Green P MacLeod CJ. SIMR: an R package for power analysis of generalized linear mixed models by simulation. Methods Ecol Evolution. (2016) 7:493–8. 10.1111/2041-210X.12504

[87] Little RJA. A test of missing completely at random for multivariate data with missing values. J Am Statist Assoc. (1988) 83:1198–202. 10.1080/01621459.1988.10478722

[88] Jakobsen JC Gluud C Wetterslev J Winkel P. When and how should multiple imputation be used for handling missing data in randomised clinical trials - a practical guide with flowcharts. BMC Med Res Methodol. (2017) 17:162. 10.1186/s12874-017-0442-1 29207961 PMC5717805

[89] John WT. The future of data analysis. Ann Mathemat Statist. (1962) 33:1–67. 10.1214/aoms/1177704711

[90] Tabachnick BG Fidell LS. Using Multivariate Statistics 7th ed. Pearson (2018).

[91] Love J Selker R Marsman M Jamil T Dropmann D Verhagen J. JASP: graphical statistical software for common statistical designs. J Statist Softw. (2019) 88:1 – 17. 10.18637/jss.v088.i02

[92] Crawford JR Howell DC. Regression equations in clinical neuropsychology: an evaluation of statistical methods for comparing predicted and obtained scores. J Clin Experi Neuropsychol. (1998) 20:755–62. 10.1076/jcen.20.5.755.1132 10079050

[93] Jacobson NS Truax P. Clinical significance: a statistical approach to defining meaningful change in psychotherapy research. J Consult Clin Psychol. (1991) 59:12–9. 10.1037/0022-006X.59.1.12 2002127

